# The impact of floating photovoltaic power plants on lake water temperature and stratification

**DOI:** 10.1038/s41598-023-34751-2

**Published:** 2023-05-16

**Authors:** Konstantin Ilgen, Dirk Schindler, Stefan Wieland, Jens Lange

**Affiliations:** 1 Fraunhofer Institute for Solar Energy Systems ISE, Heidenhofstr. 2, 79110 Freiburg, Baden-Württemberg Germany; 2 Hydrology, Faculty of Environment and Natural Resources, University of Freiburg, Freiburg, Baden-Württemberg Germany; 3 Environmental Meteorology, Faculty of Environment and Natural Resources, University of Freiburg, Freiburg, Baden-Württemberg Germany

**Keywords:** Limnology, Renewable energy

## Abstract

Floating photovoltaics (FPV) refers to photovoltaic power plants anchored on water bodies with modules mounted on floats. FPV represents a relatively new technology in Europe and is currently showing a rapid growth in deployment. However, effects on thermal characteristics of lakes are largely unknown, yet these are crucial for licensing and approval of such plants. Here, we quantify FPV impacts on lake water temperature, energy budget and thermal stratification of a lake through measurements of near-surface lateral wind flow, irradiance, air and water temperatures at one of the largest commercial German facilities, situated on a 70 m deep dredging lake in the Upper Rhine Valley, South-West Germany. Underneath the FPV facility, a 73% reduction in irradiance on the lake surface and an average 23% reduction in near-surface wind speed at module height are detected. A three month data set is then used to set up the General Lake Model and simulate scenarios of different FPV occupancies and changing climatic conditions. We observe that a lake coverage with FPV result in a more unstable and shorter thermal stratification during summer, which could mitigate the effects of climate change. The reduction of water temperatures follows a non-linear relationship with increased FPV occupancy. A sensitivity analysis showed that an increased wind reduction by FPV can have a considerable impact on the thermal properties of the lake. However, measurements only suggest small deviations with regard to the thermal properties of the investigated lake. These findings can be used in approval procedures and allow for a more accurate assessment of environmental impacts of future installations.

## Introduction

To combat climate change, more and more countries strive for greenhouse gas neutrality. To achieve this goal, renewable energies must cover energy demand as far as possible, which goes hand in hand with a massive expansion of installed photovoltaic capacity. Based on various transformation scenarios, the Fraunhofer Institute for Solar Energy Systems ISE estimates that an expansion target of 300 to 450 gigawatt-peak (GWp) of photovoltaics (PV) for Germany is plausible for the target year of 2040 for the energy sector^1^. This would require an average annual net addition of 13 to 21 GWp, plus replacement installations. Annual net addition in 2013–2018 averaged 1.9 GWp, whereas in 2020, 4.9 GWp were installed^2^.

In Germany, conventional PV ground-mounted systems are mainly permitted on areas along railroads or highways. Since 2017, it is possible to tender for bids on grassland and farmland. However, the former is almost exhausted in Germany, and per capita, arable land has declined sharply worldwide in recent years^3^. To address land scarcity and waning local acceptance of ground-mounted PV systems, innovative concepts such as integrated photovoltaics have increasingly come into focus in recent years. Integrated photovoltaics refers to area-neutral PV power generation such as agrivoltaics (APV), building-integrated PV (BIPV), PV along traffic routes (RIPV) and vehicle-integrated PV (VIPV), all of which allow for a dual use of module-covered areas^4^.

Another type of integrated photovoltaics is floating PV (FPV), where PV modules are placed on floating substructures on off- or onshore water bodies, mitigating competition for land usage. The technical FPV potential is estimated at 400 GWp worldwide, which is associated with using 1% of the world's surface waters^5^. In Germany, the technical potential on artificial water bodies alone is estimated at 44 GWp^4^. The technology is currently most widespread in the Asian region^6^, while FPV systems could produce almost 10% of the current US electricity demand by covering just 27% of suitable water bodies^7^.

Main current FPV challenges include mooring, fluctuating water levels, wind and wave loads, and increased corrosion through saltwater exposure in offshore FPV^5^. However, FPV also offers several advantages compared to conventional PV systems such as less costly site preparation and maintenance, synergy effects from coupling FPV with other installations (e.g., adjacent wind and hydropower plants) and reduced evaporation^8,9^. In addition, the cooling effect of water can increase yields by up to 15% compared to comparable ground-mounted PV systems^10–12^.

Even though many megawatt-scale FPV plants are currently in service around the world, the effect of FPV on a water body’s hydrological and ecological status are largely unknown^13,14^. As a consequence, several studies have recently been initiated to explore environmental impacts in more detail. Different FPV prototypes and their influence on electricity yield, water quality, and ecology were analysed in a study. They indicated that anoxic conditions were not favored below FPV, whereas hypoxic conditions, classified here at concentrations < 6 mg/l dissolved oxygen, occurred about 80% more frequently. Furthermore, a threefold lower biomass accumulation of submerged macrophytes below the FPV plant was detected at the studied lake in the Netherlands^12^. Other authors simulated the installation of a FPV system on Windermere (England). They were using the MyLake model to simulate how a potential FPV facility will affect the lake's water temperature and evaporation. It was simulated that at high FPV occupancies surface water temperature reductions of up to 8 °C as well as a stratification period reduced by up to 200 days can occur. A possible gain in water as a result of dew formation was also determined in the analysis of evaporation^15^.

In another study, the effects of FPV on the water quality were investigated at the Bomhofsplas PV park (27.4 MWp, Netherlands). Here, measurements of water temperature and dissolved oxygen were carried out using an underwater drone. In winter, an average of 1.1 mg/l lower oxygen concentrations could be measured under FPV, while these were 1.7 mg/l lower in summer^16^.

However, there are hardly any studies that sufficiently describe the impact of FPV on water temperature and stratification using stationary monitoring concepts with high temporal and spatial resolution data. In addition, the models used in other studies have not been fed with measured data that adequately describe the impact of FPV, but are mostly based on assumptions. The impact of FPV under changing climatic conditions as a result of climate change has hardly been investigated so far and should be the subject of FPV research in order to shed more light on the long-term perspectives of the technology.

In this study we investigate the impact of a FPV system on near-surface lateral wind flow, irradiance, surface water temperature and energy balance of a lake using extensive meteorological and hydrological measurements at one of the few commercial FPV power plants in Germany. The data recorded at the 749 kWp system on Lake Maiwald near Renchen/Baden is analysed with respect to thermodynamic processes, extreme weather events as well as to resulting thermal stratification, and also serves as a basis for hydrological modelling.

On the one hand, FPV reduces irradiance on the water surface and directly affects the energy balance of the covered lake, which could lead to more unstable stratification^9,12^. On the other hand, FPV is expected to affect wind shear at the water surface and reduce near-surface wind speed resulting in a more stable stratification due to reduced mechanical mixing^15^. Consequently, FPV installation can affect a lake's thermal stratification in various and competing ways.

The net effect on lake stratification and mixis is the key aspect of this work, with the FPV system at Lake Maiwald as a case study.

Furthermore, we use a lake water balance and stratification model to determine how extreme climatic conditions, i.e., hot and dry summer periods, modify FPV impacts. This allows to assess whether local effects of climate change are promoted or mitigated by FPV. By modelling, the influence of different FPV occupancies can also be considered.

## Materials and methods

### Study site and measurements

**Figure 1:**
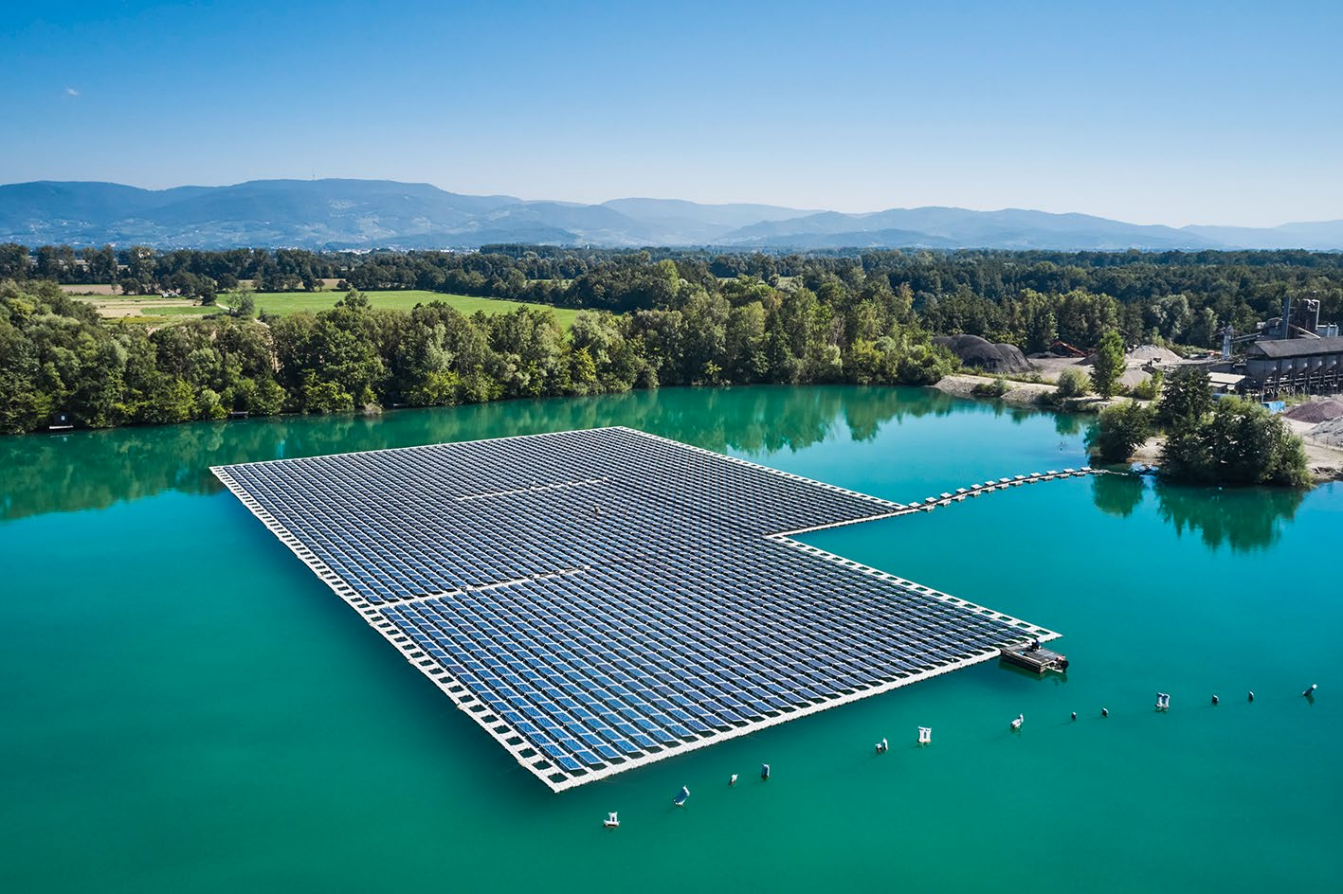
Floating PV power plant installed on Lake Maiwald with a net capacity of 749 kWp near Renchen/Baden. Photo: Jan Oelker.

Lake Maiwald (lat. 48.645, lon. 7.986) is located in south-west Germany within the Upper Rhine Valley between the Black Forest in the east and the river Rhine in the west. Lake Maiwald is one of several dredging lakes in the Upper Rhine valley which are actively excavated. During the three-month observation period from July 17, 2021 to October 15, 2021, dredging occurred in the western portion of the lake, while the FPV facility is located in the southeastern part of the lake. The system was completed in July 2019 and consists of 2300 modules, which are arranged on an area of approx. 7700 m2 (Fig. 1). The lake has a total area of 37 ha and an average depth of 24 m. The maximum depth is 70 m and overall lake volume amounts to 8.8 × 10^6^ m^3^. In the given latitude, lakes with such a depth are classified as dimictic and establish a stable thermal stratification during summer^17,18^.

For the meteorological measurements, a weather station with an integrated CR1000 data logger is installed in the center of the FPV system. The height of the station is 2 m above the water surface. It is equipped with a resistance thermometer with capacitive humidity sensor to measure the air temperature (°C) and relative humidity (%). Net radiation (W m^−2^) is measured with a CNR1 net radiometer consisting of two pyranometers and two pyrgeometers (Kipp & Zonen, The Netherlands). The pyranometers measure incoming and surface-reflected shortwave radiation (0.3 to 2.8 µm), while the pyrgeometers measure incoming and outgoing longwave radiation (4.5 to 42 µm). The accuracy for daily totals is ± 10%. A 2D ultrasonic anemometer is attached to the top of the weather station at 2 m. It is used to measure wind speed (m s^−1^) and wind direction (°). The accuracy of the wind direction measurement is ± 3°. For wind speed measurements it is ± 0.3 m s^−1^ or ± 3% in the range 0–35 m s^−1^.

**Figure 2:**
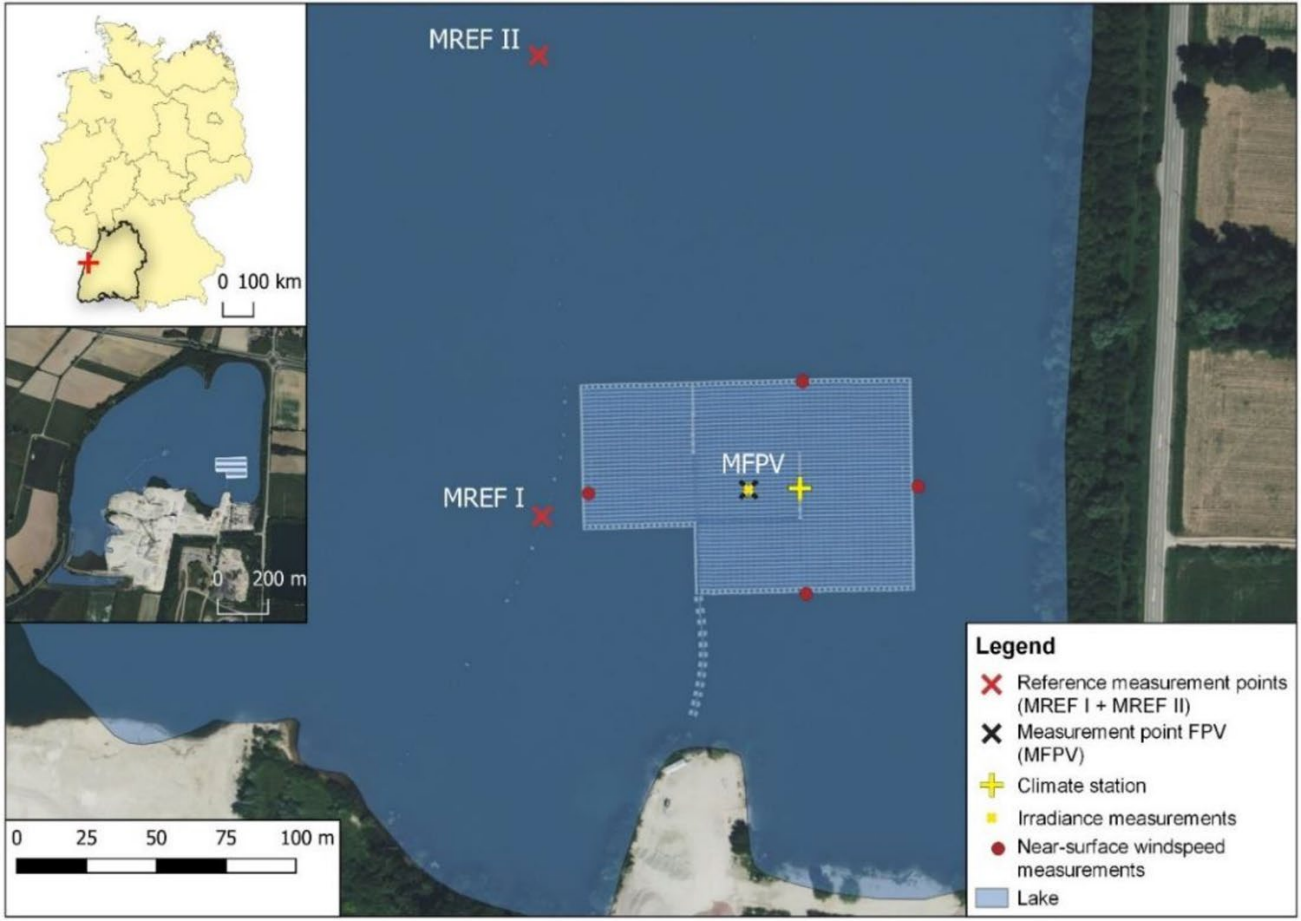
Location of the study site and measurement points.

Since there is a different surface roughness in the FPV area due to the elevated modules compared to the water surface, the near-surface airflow could be affected differently depending on the module tilt angle. For water surfaces, the roughness length z_0_ is significantly lower (z_0_ = 0.0002 m) than for densely populated or forested areas (z_0_ = 1.6 m)^19^. As a result of the FPV power plant and its tilted modules, the roughness length in the area of the facility is increased, which is expected to reduce wind shear at the water surface. Four small wind transmitters (Adolf Thies GmbH & Co. KG, Germany) are placed on each side at the edge of the power plant to investigate the potential impact of FPV on the near-surface wind speed. The height of the measuring instrument corresponds to the maximum module height above the water level. The measuring devices are placed opposite to each other so that the weather station represents the intersection of the east–west and north–south straight lines between the devices (Fig. 2).

In addition to the meteorological measurements, depth gradients of water temperature are measured inside the lake, which is considered a fundamental variable in limnology and often used to infer interactions for the entire system^20,21^. HOBO pendant temperature/light 64K data loggers (Onset, USA) continuously measure water temperature in hourly intervals and are attached to a 10-m line of insulated wire rope at 50 cm intervals. The measurement accuracy of the HOBOs is ± 0.53 °C. To assess impacts of the FPV system, measurements beneath the system (MFPV, Fig. 2) are compared to measurements at two reference sites. Reference measurement site I (MREF I) is located a few metres west of the FPV system, whereas reference measurement site II (MREF II) is about 120 m north of the northern edge.

Based on the measured water temperature, the thermoclines for the respective measuring points can be calculated. The thermocline depth was defined as the planar separation of the surface mixed layer from the bottom stagnant layer. The specific depth of this planar thermocline was quantified as the depth of the maximum density difference over the vertical axis where the minimum water temperature was above 4 °C and the density difference between the surface and bottom layer was above 0.1 kg m^−3^, signaling stratified conditions^22^. In addition, correlations between depth and mean water temperature differences, and depth and the standard deviation of mean water temperature differences are calculated. To quantify the statistical validation of the spatial impact of FPV and water temperature fluctuations, a two-sided t-test is performed.

### Energy balance

A method for determining the change in heat storage using data on water temperature is applied to determine the energy balance^23^. It is calculated in one hour time steps for each measurement point and compared between FPV and reference points. The calculation is made per square metre for a 10-m deep water column, since this corresponds to the range of the water temperature measurement. The heat storage change ∆G (J m^−2^) is calculated using the following equations:

1 $$\Delta G=G\left({t}_{2}\right)-G({t}_{1})$$

with

2 $$G\left(t\right)={C}_{PW}{\int }_{z={Z}_{0}}^{z={Z}_{max}}{\rho }_{W}\left(z,t\right) {\Delta T}_{W}\left(z,t\right) dz$$

where C_PW_ is the specific heat capacity of water (4184 J kg^−1^ K^−1^), ρ_W_ the density of water (kg m^−3^) at depth *z* (m) and time t (s), and ∆T_W_ the water temperature difference (K).

### General lake model

To extrapolate the observed impacts of the FPV system, the General Lake Model (GLM, version 3.1) is applied^24^. The GLM is a vertical 1D model that represents the hydrodynamic processes of lakes, water reservoirs or wetlands and uses a flexible Lagrangian grid, with the thickness of each layer changing dynamically as a function of the respective water density. Surface mixing processes are calculated using an energy balance approach that compares the available (turbulent) kinetic energy with the internal potential energy of the water column^22,25^. A detailed manual describing the algorithms used in the model is available^25^.

To investigate the responses of lakes in the U.S. state of Wisconsin to regional climate change, over 2000 lakes were modelled in a study^26^. In another study, the biochemical-ecological model component (AED_2_) of the GLM and a hydrological catchment model (PIHM-Lake) were used to simulate the annual variability of anoxic conditions in eutrophic Lake Mendota^22^. To evaluate the performance of GLM, a study simulated 32 lakes from the Global Lake Ecological Observatory Network (GLEON)^27^.

**Figure 3:**
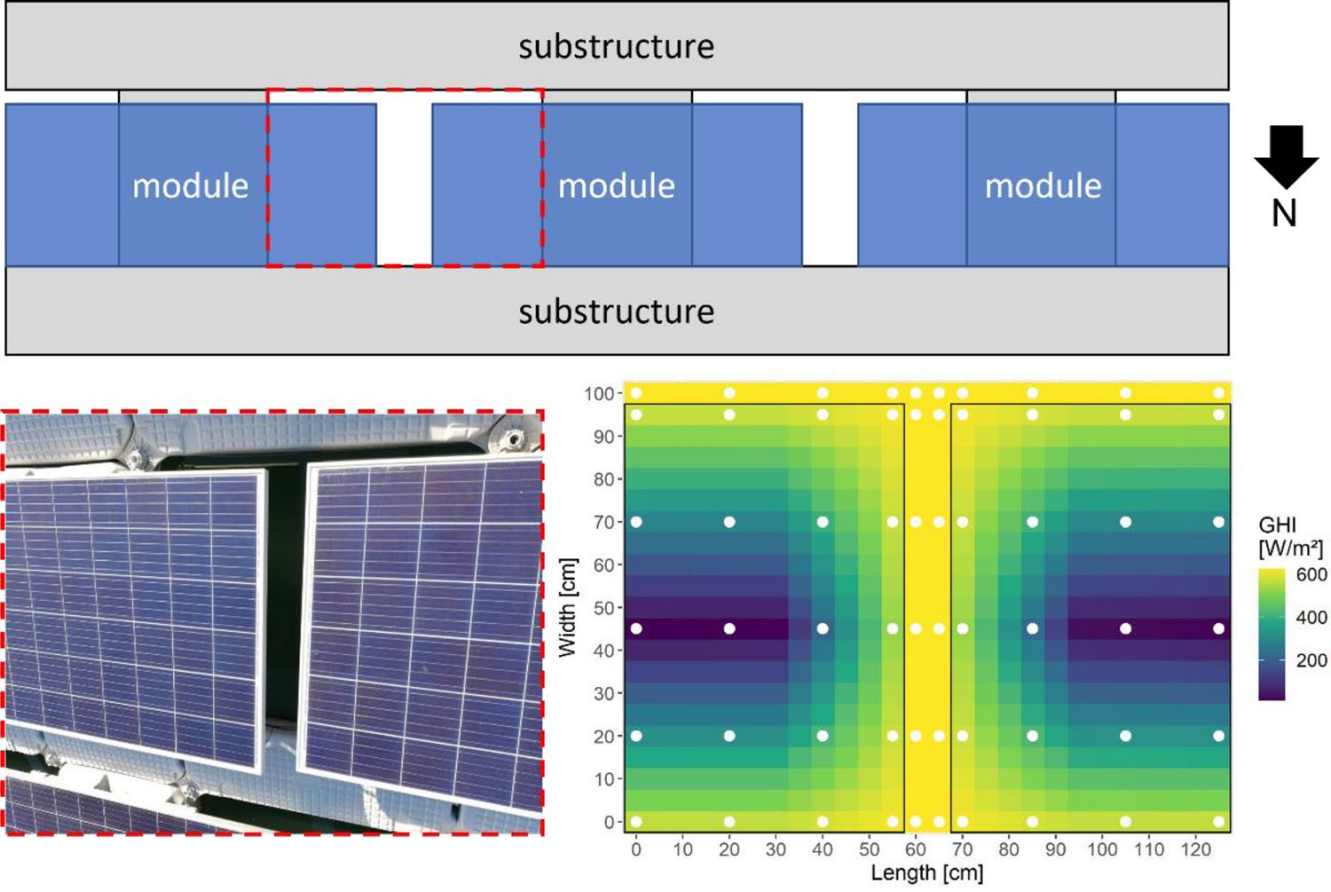
Top view of a module row section within the FPV system (top) and results of the measurements shown using systematic interpolation based on measurements of irradiance above and below the modules (bottom right). The measurements are taken below the modules at the points marked in white.

It can be assumed that the parameters mostly affected by FPV are solar irradiance into the lake and near-surface wind speed^15^. Therefore, the present analysis concentrates on these two forcing variables. The area covered by FPV mainly consists of three surface types, namely modules, water surface and substructure (floats). To determine the irradiance reduction caused by FPV, measurements of global horizontal irradiance (GHI) above and below the modules are taken, and systematic interpolation is used to determine the proportion that still enters the lake through the system. The measurements are done in a measurement campaign and took place every two and a half weeks. Hence, five individual measurement procedures are carried out during the observation period. The irradiance above and below the modules is measured using a CS300 pyranometer (absolute accuracy: ± 5% for daily total radiation). The measurements are carried out below the modules at the white points marked in Fig. 3. Measurements above the FPV system are made at the top of the module. In contrast to the measurements below the modules, there is hardly any variation in the measured values above system. The impact of the shading through the modules is shown in the irradiance matrix, where the averaged values from the individual measurements are systematically interpolated. The measured values range from 11.5 to 625 W m^−2^. This considerable variability may highlight the need for spatially distributed measurements as part of measurement campaigns. A continuous measurement using a single pyranometer under the modules could possibly only capture a selected portion of the radiation reduction, depending on placement. However, it should be considered that spatially resoluted measurement in campaigns may not be fully capable of capturing the entire diurnal variations. Assumptions are made that no irradiation can enter the lake through the substructure due to the closed design and the high albedo. Irradiation hitting the water surface between the modules or in the open water areas within the system remains unaffected.

Accounting for the geometric layout of the FPV system, the following equations result for the fraction of irradiance that enters the lake below the system (λ) and respectively the fraction of irradiance that is blocked by the system (${sw}_{r}$):

3a $$\lambda =\frac{\overline{{R }_{M}}{A}_{M}{n}_{M}+{A}_{R}{R}_{s}}{{A}_{FPV}{R}_{s}}$$

3b $${sw}_{r}=1- \lambda$$

where $\overline{{R }_{M}}$ (W m^−2^) is mean GHI measured between and below the modules, A_M_ the typical size of openings in the floating structure (red dashed rectangle in Fig. 3) (m^2^), n_M_ the number of modules, A_R_ the area of open water within the power plant area (m^2^), R_s_ the mean GHI above the modules (W m^−2^) and A_FPV_ the total power plant area (m^2^).

**Figure 4:**
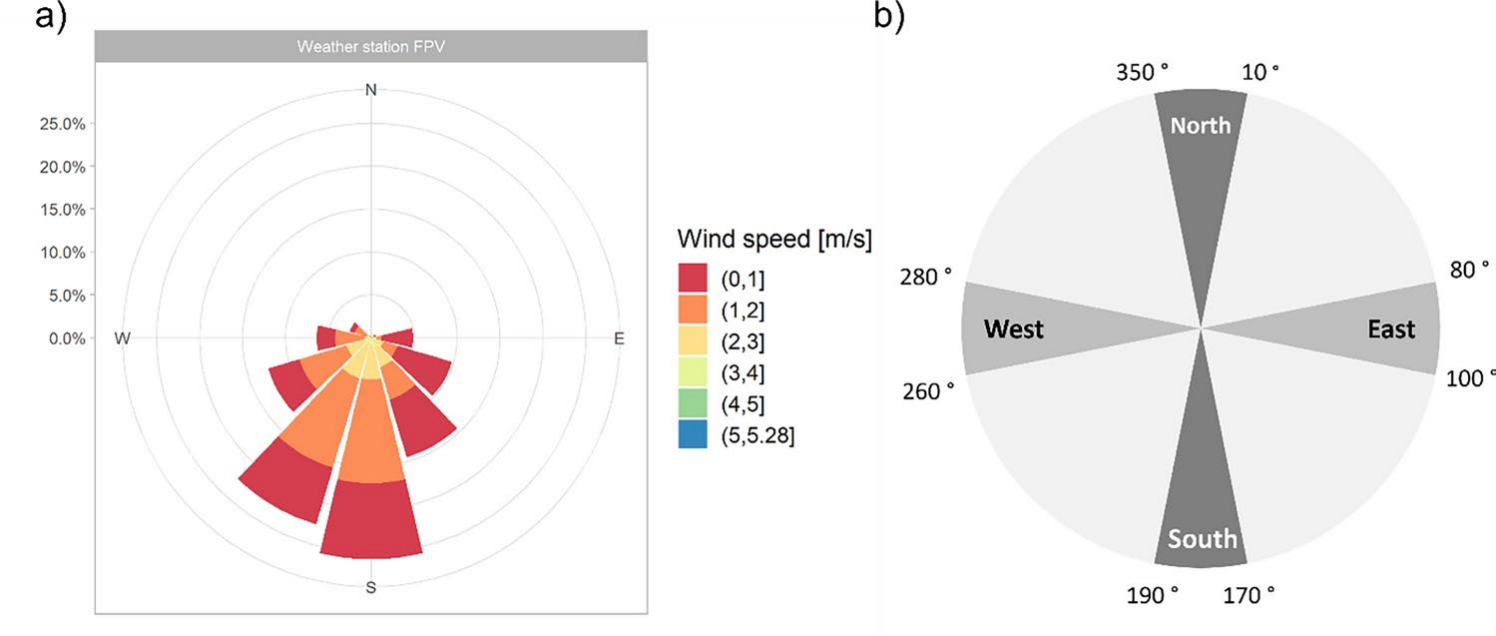
(**a**) Measured wind speeds and directions at the weather station, and (**b**) definition of the effective wind direction for filtering the measured wind speeds of the wind transmitters.

To incorporate the impact of the system on the near-surface lateral wind speed into the model, the data generated by the small wind transmitters are used. Here, data from the opposing measuring devices are compared with each other. The wind direction and speed measured by the weather station at 2 m height are depicted in Fig. 4a. Since the transmitters can only measure the wind speed, the station's measured wind directions are used to filter the data from the small wind transmitter based on the effective wind direction. The effective wind direction defines the wind directions north, east, south, and west corresponding to the transmitter positions, each with a tolerance angle of ± 10° (Fig. 4b). Only wind speed within this range are included in the further analysis to ensure that the wind flow is along the horizontal and vertical axes of the FPV system. For example, if the wind is coming from the south, the difference between the readings from the sensor on the southern edge and the one on the northern edge determines the impact of FPV. Most of the transmitter measurements are assigned to the effective wind direction 'South'. For the effective wind direction 'North', no measurements can be assigned. The calculated percentage of wind reduction in each direction is then weighted by the number of measurements in each direction. The mean of the respective weighted percentages represents the overall reduction in near-surface lateral wind speed due to FPV (${w}_{r}$).

Since the GLM simulates the entire lake system and the FPV power plant only covers a part of the lake, these factors must be area-weighted, resulting in the so-called forcing variables. The area-weighted forcing variable for the irradiance ($s{w}_{aw}$) and the near-surface lateral wind speed (${w}_{aw}$) is calculated as

4a $$s{w}_{aw}={sw}_{f}\left(1-\frac{{A}_{FPV}}{{A}_{S}}{sw}_{r}\right)$$

4b $${w}_{aw}={w}_{f}\left(1-\frac{{A}_{FPV}}{{A}_{S}}{w}_{r}\right)$$

where $s{w}_{f}$ is the uncalibrated factor for short wave radiation (unitless, in this case $s{w}_{f}=1$), ${w}_{f}$ the respective uncalibrated factor for wind (unitless, in this case ${w}_{f}=1$), A_FPV_ the total power plant area (m^2^), A_s_ is the lake surface area (m^2^), ${sw}_{r}$ the overall reduction in irradiance and ${w}_{r}$ the overall reduction in near-surface lateral wind speed due to FPV (unitless).

In a first step, GLM is applied at MREF II and calibrated to measured water temperature. In a second step, the FPV impact is modelled by adjusting the forcing variables based on measured reductions of irradiance and near-surface wind speed, which is validated by measured lake temperature. In a third step, simulations of different FPV occupancies (15%, 50% and 90%) are performed. Thereby, a 90% occupancy represents the maximum technically feasible occupancy^4^. The impact of FPV under changing climatic conditions is investigated by simulating the extremely hot and dry year 2018 where an air temperature anomaly of 2.3 K was recorded (compared to the climate reference period 1961–1990). To quantify the corresponding change in stratification stability, the Schmidt stability is used. Schmidt stability (S_T_ in J m^−2^) represents a stability index that expresses the amount of energy needed to mix the entire water column to uniform temperatures without affecting the amount of internal energy, while the internal energy (E in J m^−2^) is the thermal energy stored in the water column^28,29^. If Schmidt stability is greater than zero, stratified conditions in the lake are to be assumed. Simultaneously, the formation of a thermocline can be assumed. S_T_ and E are quantified using the R package rLakeAnalyzer^30^ as

5 $${S}_{T}=\frac{g}{{A}_{s}}\underset{{z}_{0}}{\overset{{z}_{max}}{\int }}{A}_{z} {\rho }_{z} \left({z}_{v}-z\right) dz$$

and

6 $$E=\underset{{z}_{0}}{\overset{{z}_{max}}{\int }}{C}_{PW} {\rho }_{z} {T}_{z} dz$$

where g is the standard gravity, A_s_ is the surface area (m^2^), z_0_ is minimum and z_max_ the maximum depth (m), A_z_ is the respective area (m^2^) at depth z, ρ_z_ is the respective density at depth z (kg m^−3^), T_z_ is the water temperature at depth z (K), C_PW_ is the specific heat capacity of water (4184 J kg^−1^ K^−1^), z_v_ is the depth of the center of volume (${z}_{v} = \frac{1}{V} \underset{{z}_{0}}{\overset{{z}_{max}}{\int }}{A}_{z} z dz$), and V is the volume (m^3^). Finally, the measurement of wind reduction by FPV is a complex measurement procedure, which can be subject to uncertainties due to technical limitations by the conditions on FPV power plants. To highlight the relevance of wind reduction by FPV and its effect on the thermal properties, we perform a sensitivity analysis.

## Results and discussion

### Water temperature and stratification

**Figure 5:**
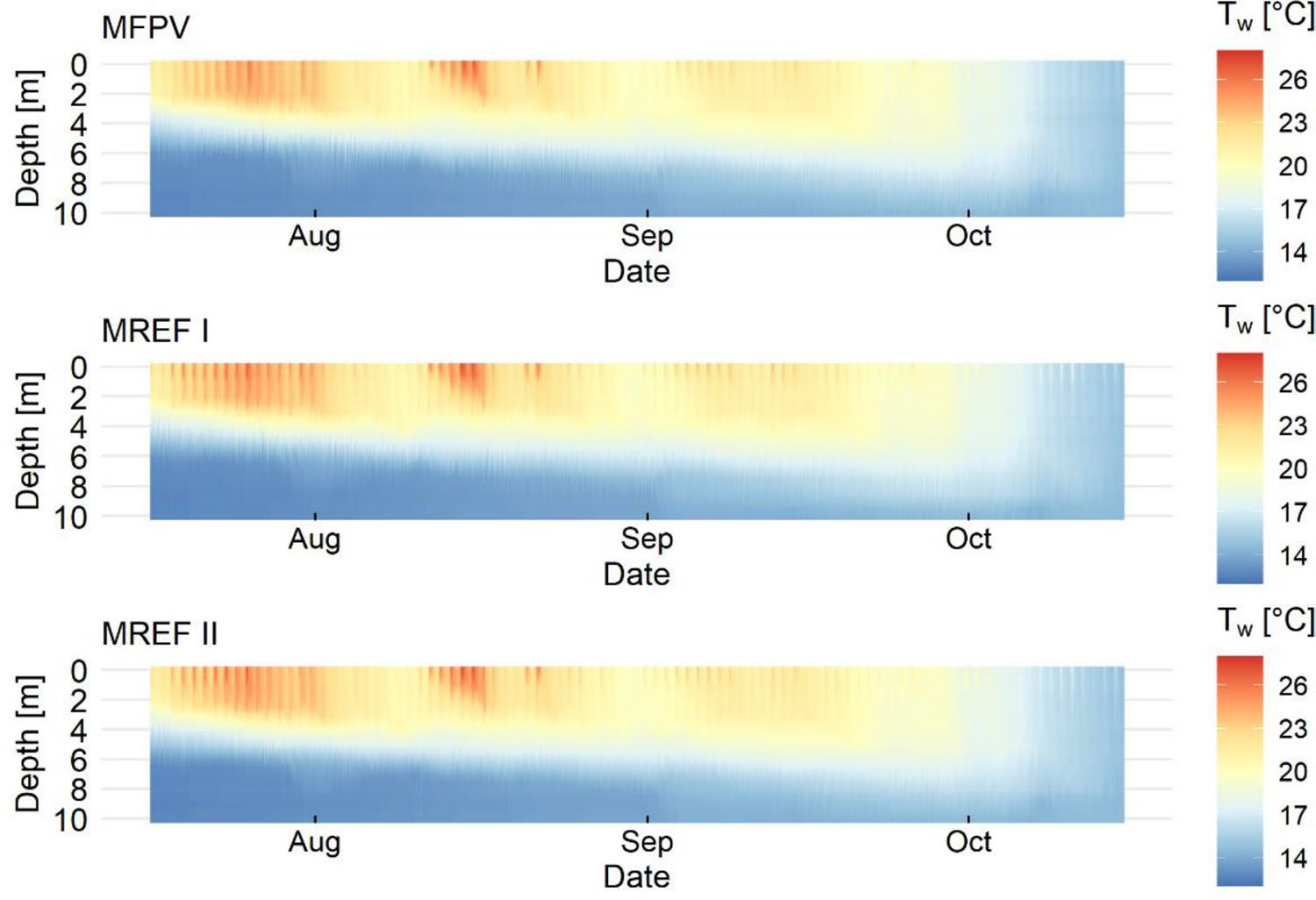
Depth profiles of lake water temperature recorded at the three measurement points.

The measured water temperature (T_w_) suggests that a stable thermal stratification builds up during summer at all three measurement points. The date of the onset of vertical mixing at the beginning of October is similar at all measurement points (Fig. 5). Data describing the water temperatures at the measurement points are available in the Supplementary Information.

**Figure 6:**
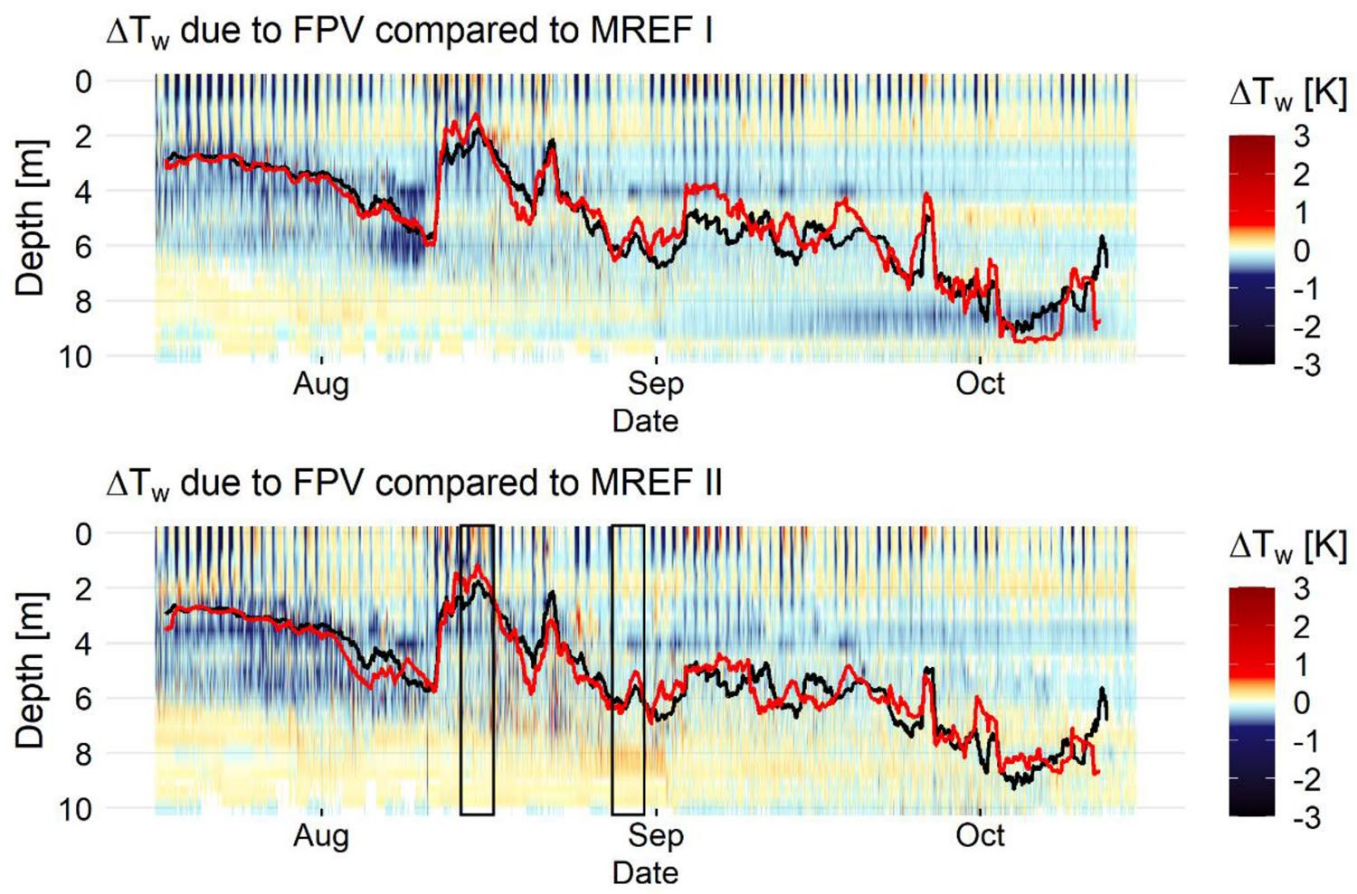
Water temperature difference (ΔT_w_) caused by FPV compared to reference measurement point I (MREF I) and reference measurement point II (MREF II). Light colors represent data within the range of measurement uncertainty, while dark colors represent measured values outside this range. The black line refers to the thermocline below FPV, the red line to the thermocline at the respective reference measuring point. The black rectangles indicate the time sections shown below in Fig. 7.

The water temperature differences (ΔT_w_) due to FPV compared to the reference measurement points are shown in Fig. 6. It can be seen that similar ΔT_w_ patterns induced by FPV can be observed when compared to MREF I and MREF II. In the depth range between 2 and 6 m, a negative ΔT_w_ due to FPV can be observed. MFPV shows higher water temperature below 6 m water depth at the beginning of the observation period compared to MREF I and MREF II. At a depth of 10 m, hardly any deviations are visible, indicated by light color areas. The depth and mean water temperature differences show a moderate positive correlation (r = 0.57).

The thermocline of FPV is described by the black line, while those of the references are indicated by the red lines. The strong offset of the thermocline in mid-August to lower water depth is due to the strong warming of the epilimnion, which consequently shifts the temperature gradient to higher water layers. The mean thermocline depth for FPV is 4.93 m with a standard deviation of ± 1.74 m, for MREF I 4.83 ± 1.81 m and for MREF II 4.94 ± 1.64 m.

Uppermost water layers show a large ΔT_w_ variability depending on the time of day. In particular, negative ΔT_w_ values can be observed in the near-surface layer indicating a cooling effect of the system. The depth and standard deviation of mean water temperature differences show a strong negative correlation (r = −0.87). The fluctuations of the water temperature differences are significantly higher in the depth range of 0 to 5 m than in the depth range of 5 to 10 m (p = 0.0007). This could indicate that the effect of FPV is mainly reflected in the upper water layers, as well as that different effects can be expected during the day and night.

However, it can be stated that most of ΔT_w_ stays within a small range of values and often do not exceed ± 0.5 K. Taking into account the measurement accuracy of the HOBOs, ΔT_w_ is less pronounced, especially in the greater depths. The strongest cooling occurs in the near-surface water layer and amounted to ΔT_w_ of up to −2.8 K compared to MREF I. Although the impact of FPV is considered the likely cause of ΔT_w_, other factors, such as combinations of surface conditions and advection or diffusion of heat from elsewhere, should be considered.

**Figure 7:**
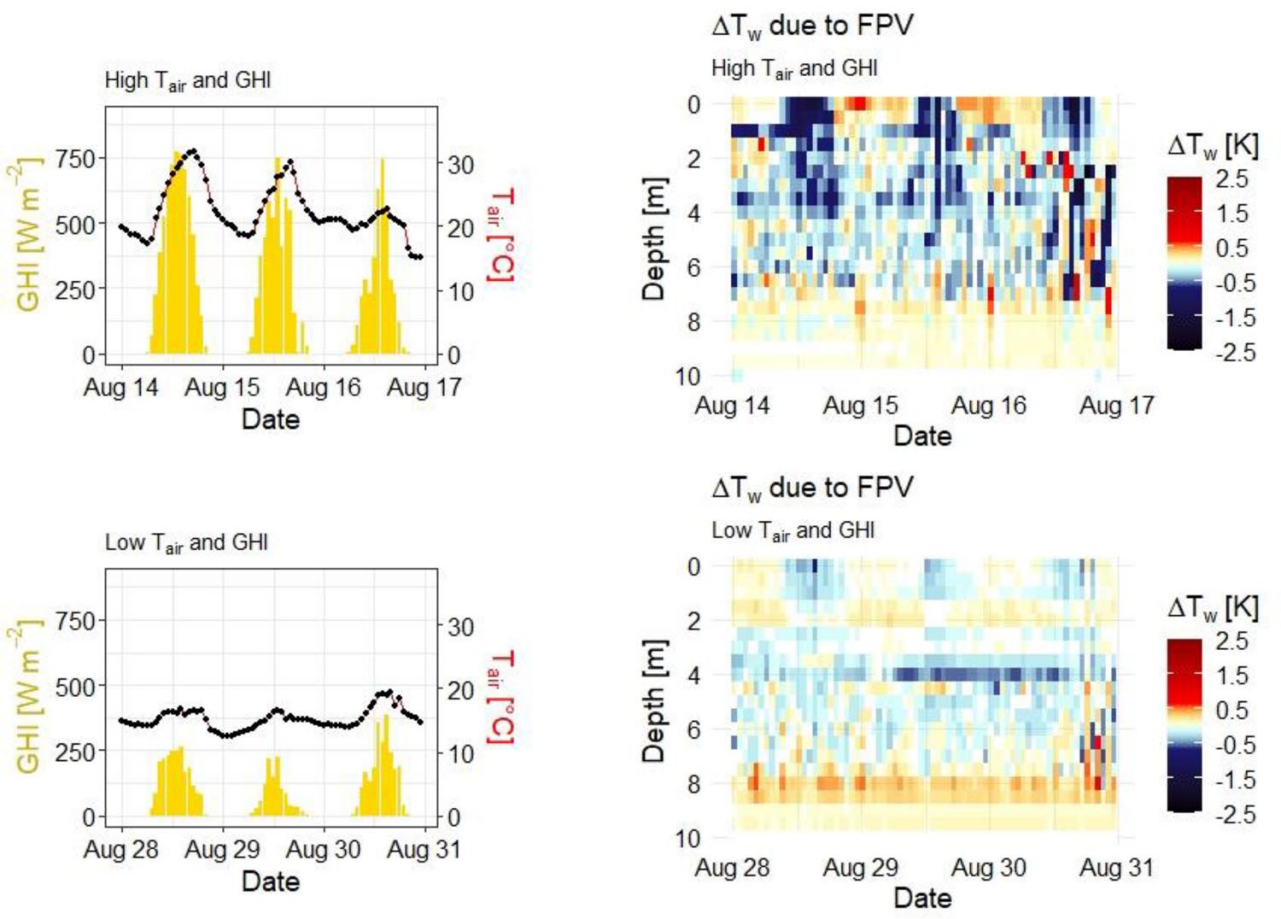
Compared variability of FPV-induced temperature difference (ΔT_w_) as a function of air temperature (T_air_) and global horizontal irradiance (GHI).

Figure 7 zooms into two periods of the study period, which significantly differ in measured GHI and air temperature (T_air_) values. In mid-August, characterised by high GHI and T_air_, negative ΔT_w_ can be seen in the uppermost water layers during the day, which indicates slower and less intense warming of the surface water due to the cooling effect by FPV. This effect is less pronounced during a cooler period during end of August when high wind speed and precipitation result in short-term changes of ΔT_w_. Then, there is also a less clear connection of negative ΔT_w_ of uppermost to deeper water layers, whereas an increased negative ΔT_w_ can be observed down to greater depths in the warmer period. While negative ΔT_w_ values are recorded during the day, positive ΔT_w_ values prevail during night until noon of the following day. The daily ΔT_w_ fluctuation ranges from + 1.4 to −2.4 K during the warm period, which is 63% larger than during the cooler period.

This effect is also shown by the values summarised in Table 1. It becomes apparent that significantly more negative ΔT_w_ values prevail during the day, which in turn indicates the cooling effect of FPV. The deviations are lower at night. From July to September, the mean ΔT_w_ remains in the negative range for the night, while in October it changes to the positive range. A trend towards lower differences was observed throughout the study period.

**Table 1:**
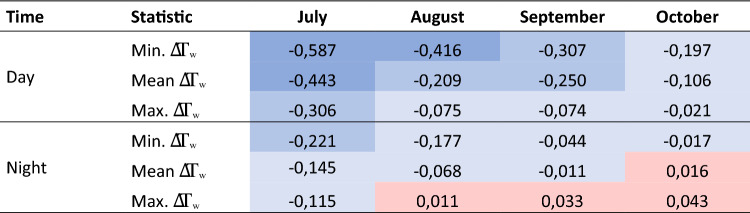
Minimum (most negative), mean, and maximum (most positive) ΔTw for every month of the observation period averaged for the day (6 am to 6 pm) and night (6 pm to 6 am).

### Energy balance

**Figure 8:**
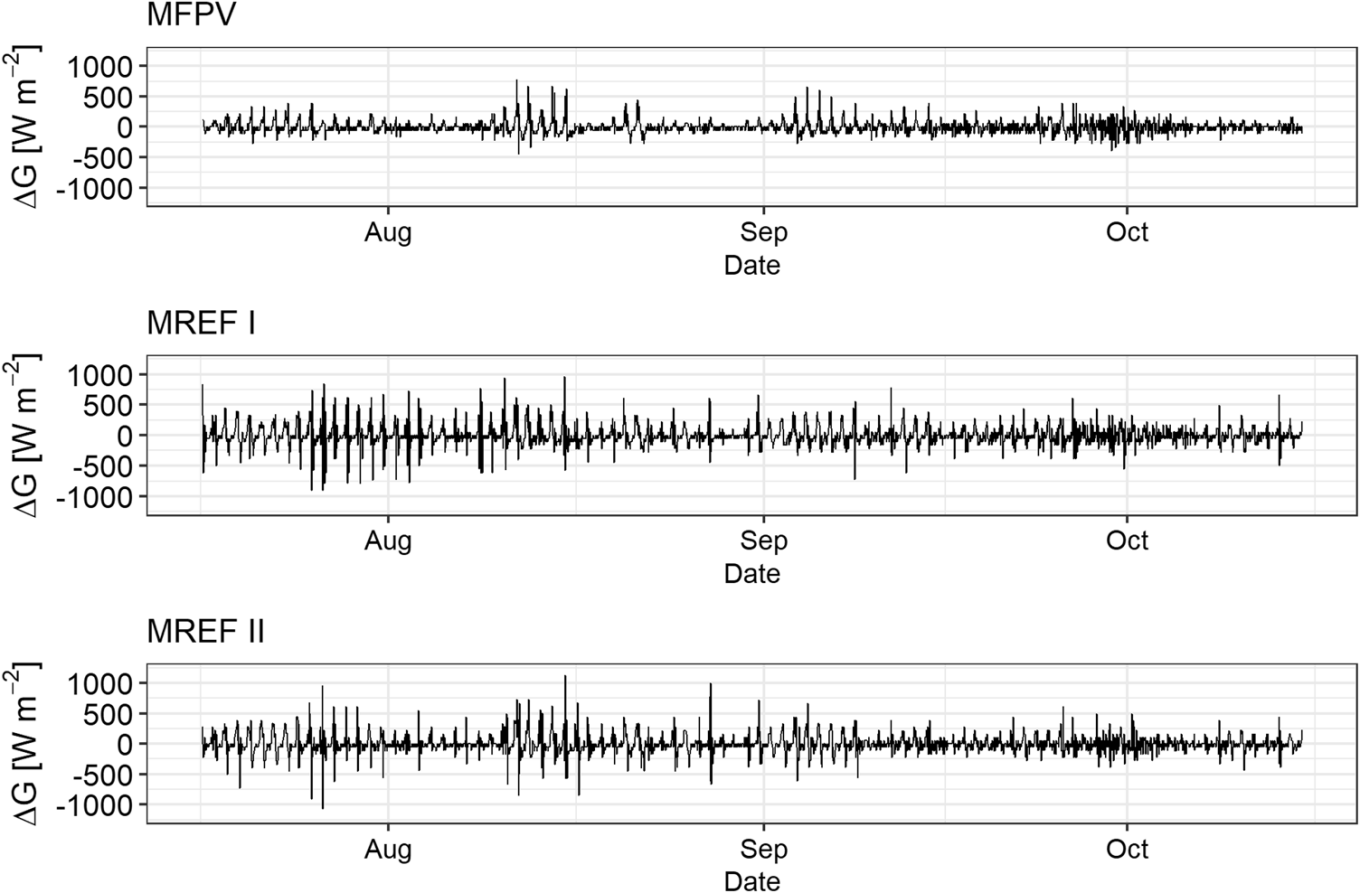
Calculated heat storage change (∆G) at the measurement points MFPV, MREF I, and MREF II.

Diurnal fluctuations of G at the two reference measurement points are noticeably higher than below the FPV system, which is reflected by larger amplitudes of ΔG (Fig. 8). The standard deviation for ΔG is ± 104.1 W m^−2^ for FPV, ± 176 W m^−2^ for MREF I and ± 162.7 W m^−2^ for MREF II. Also mean G during the study period is on average 2.2% lower at MFPV, compared to the reference measurement points.

**Figure 9:**
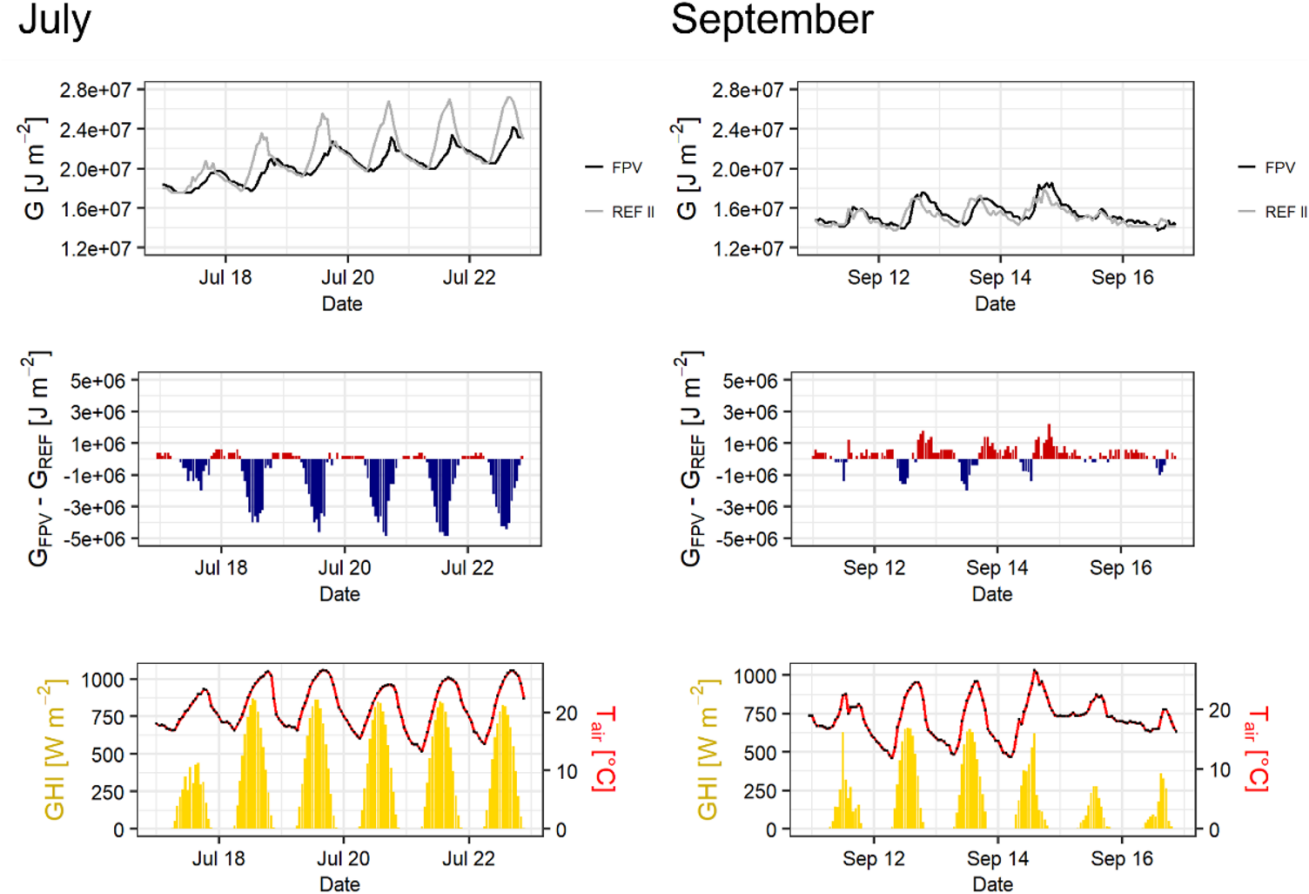
Comparison of the heat storage (G) between MFPV and MREF II based on different representative sections within the investigation period (top). Additionally plotted is the difference due to MFPV compared to the MREF II (G_FPV − _G_REF_) (middle) and corresponding GHI as well as T_air_ (bottom).

In Fig. 9, two characteristic periods from the G time series are exemplified. In July, G at MREF II is significantly higher than at MFPV, paticularly during the day. This is reflected in negative differences between G of MFPV (G_FPV_) and MREF II (G_REF_). Furthermore, a delayed increase and a delayed maximum occur at MFPV. At both sites high T_air_ and GHI prevail during the day. At night, MFPV shows higher G, since MREF II is subjected to a sharp drop of the high values during the day.

In September, T_air_ is still at a similar level compared to previous periods, while GHI is lower and T_air_ differences between day and night are smaller. G is lower and shows accentuated short-term variability, particularly towards the end of this period. Now, positive differences between MFPV and MREF II occur more frequently and exceed negative ones.

For the observation period, both the minimum and the maximum of the heat storage are delayed on average by two hours for FPV. The average daily minimum of heat storage is 13.92 megajoule per square metre (MJ m^−2^) for FPV and 13.82 MJ m^−2^ for MREF II. This is 0.1 MJ m^−2^ higher for FPV. In contrast, the average daily maximum for FPV (16.05 MJ m^−2^) is 0.69 MJ m^−2^ lower than for MREF II (16.74 MJ m^−2^).

### Hydrological modelling

The GLM is calibrated to the measured water temperatures of MREF II. The Root Mean Square Error (RMSE) of the calibration period is 0.79 °C along with Pearson’s r of 0.98 and Nash–Sutcliffe efficiency (NSE) of 0.96. For the total time period RMSE is 0.72 °C. By adjusting the forcing variables the FPV effect is represented based on measured reductions of irradiance and near-surface wind speed and validated by measured water temperatures which results in a RMSE of 0.65 °C.

**Figure 10:**
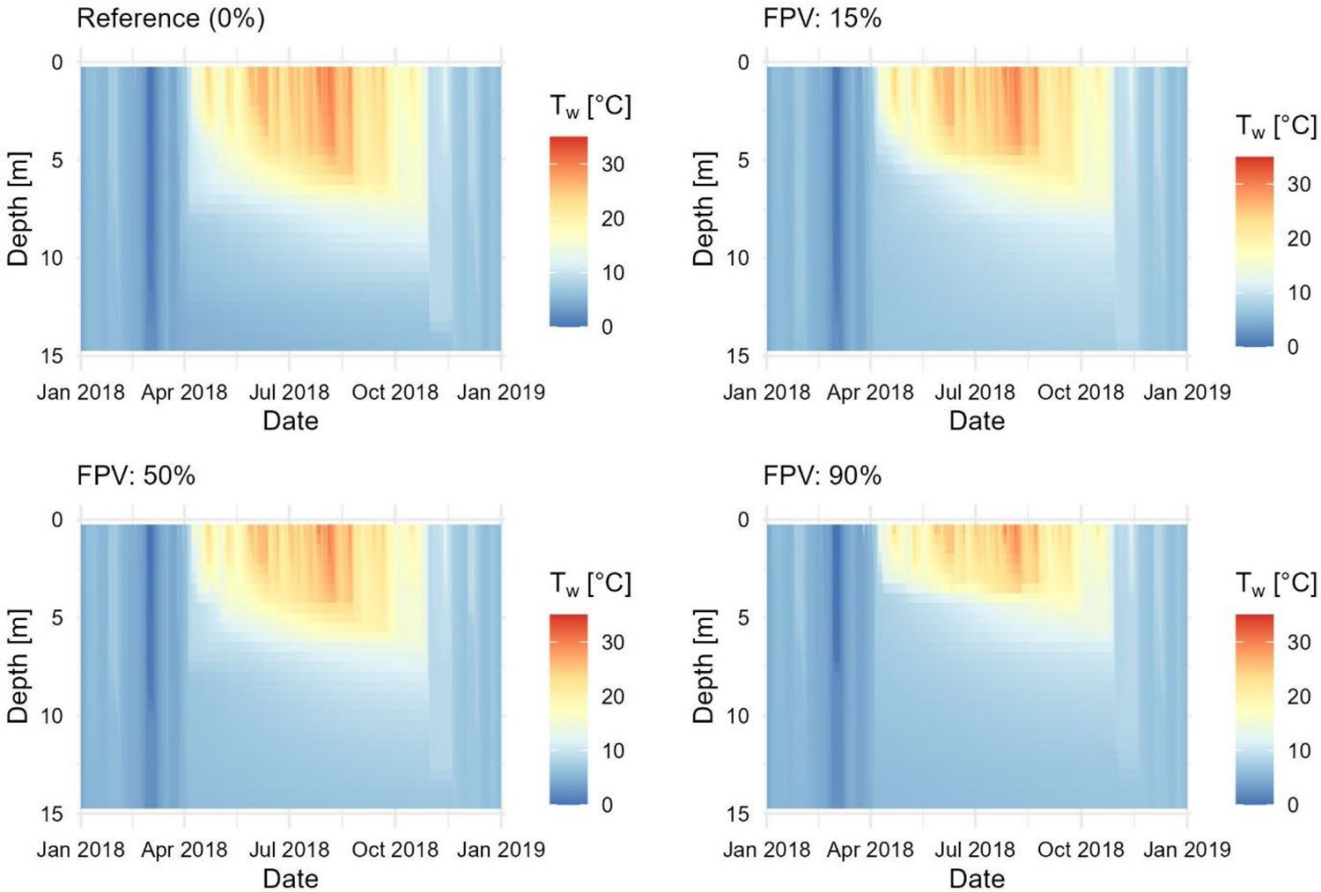
Comparison of water temperature (T_w_) from simulated FPV occupancies of 15%, 50%, and 90% with MREF II.

To illustrate FPV impact under the extreme hot and dry climatic conditions of 2018, T_w_ profiles of three simulated scenarios with 15%, 50% and 90% FPV occupancy are compared with MREF II (Fig. 10). The simulations were carried out taking into account the measured irradiance reduction of 73% and wind reduction of 23%. It can be seen that the thermal stratification forms during summer under all scenarios but becomes more unstable with increasing FPV occupancy. The stratification period starts and ends simulatenously at an area coverage of 15% compared to MREF II. In the 50% scenario, the stratification period is reduced by one week. The 90% scenario is characterised by a two weeks shorter stratification period compared to MREF II. It should be noted that due to the one-dimensionality of the GLM, the results should be considered as an averaged impact on the entire lake system.

**Figure 11:**
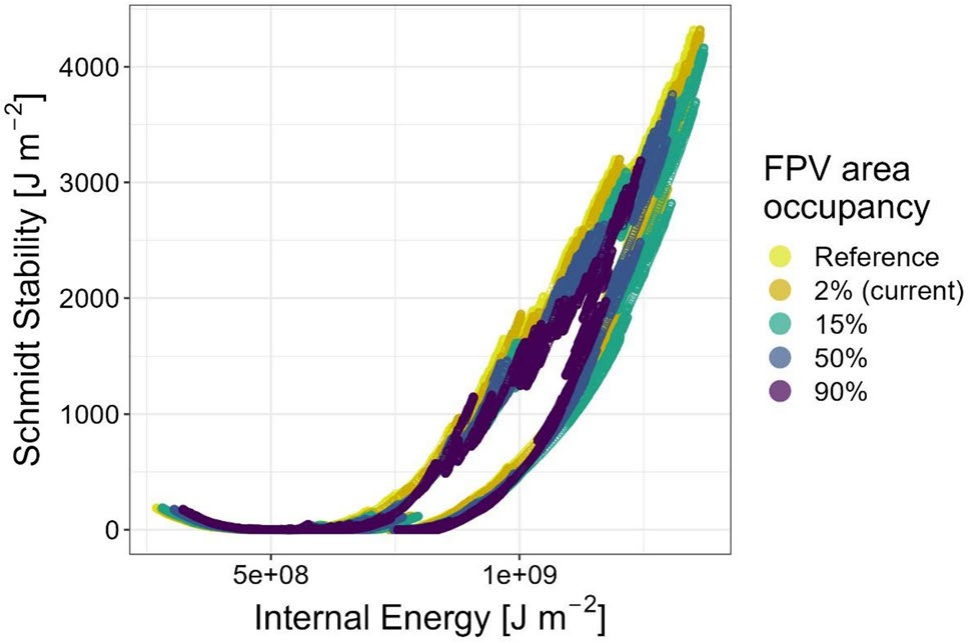
Schmidt stability (S_T_) and the internal energy (E) of Lake Maiwald as a function of simulated FPV occupancies. The simulation results of different FPV occupancies are shown on the basis of the modelled year 2018. The reference refers to the thermal stability of the lake without the impact of a FPV system. An FPV occupancy of 2% represents Lake Maiwald with the existing FPV system.

The differences in surface water temperatures are also reflected in the stability of thermal stratification expressed by Schmidt stability (Fig. 11). Note that these modelling analyses refer to the calendar year 2018, while the previous observed data results are from summer 2021.

The relationship between S_T_ and E extent exhibited a clockwise hysteresis. This can be explained, for example, by the fact that similar values for S_T_ can be observed in early summer and autumn, since the stratification builds up in early summer due to increasing warming of the surface water, while it decreases in autumn due to cooling. However, in spring and winter, there is hardly any energy transfer into the lake due to the lower air temperatures and irradiance, which results in lower E with the same S_T_ in early summer compared to autumn. In autumn, a strong energy input has already taken place in summer, so that despite increasing dissolution of the stratification, higher values for E continue to prevail there. In summer, there are the highest values for S_T_ and E due to the strong energy input and warming of the surface water layer.

The impact of different FPV occupancies on indicators describing the thermal properties of the lake for the year 2018 can be observed in Table 2. This shows that the FPV occupancy of 2% would lead to a slightly increased thermal stability compared to the reference. At higher FPV occupancies, like 50% and 90%, there is a decrease in all variables. However, a stable stratification is formed in the lake for all observed FPV occupancies in summer 2018. The more unstable thermal properties indicate that FPV could counteract a more stable and longer stratification that might be induced by climate change^31^.

**Table 2:** Impact of different FPV occupancies on the thermal properties of Lake Maiwald in the simulated year 2018. ﻿The simulation considered a irradiance reduction of 73% and a wind reduction of 23%.

FPV occupancy (%)	Mean thermocline depth (m)	Mean Schmidt stability (J m^−2^)	Mean internal energy (J m^−2^ × 10^8^)	Mean surface water temperature (°C)
0	7.3	1320.0	9.52	15.2
2	7.6	1342.7	9.82	15.2
15	7.4	1279.3	9.69	15.1
50	6.5	1129.3	9.44	14.8
90	5.0	854.5	8.91	14.4

In the following, uncertainties in the measurements are analysed. In particular, the measurement procedure for quantifying wind reduction may be subject to uncertainties. In order to measure the wind reduction by FPV as precisely as possible, several measurements at various heights at several locations on the power plant would be conceivable. This would allow the influence of FPV on the logarithmic wind profile to be estimated. However, since this method is technically limited by the complexity of the FPV system design and the sensors itself, we decided to measure both inside and outside the boundary layer of the FPV system^32^. This was done by measuring the near-surface wind speed at module height on the edges of the system. However, in order to show the relevance of the wind reduction with respect to the impact on the thermal properties of the lake, we have performed a sensitivity analysis.

**Figure 12:**
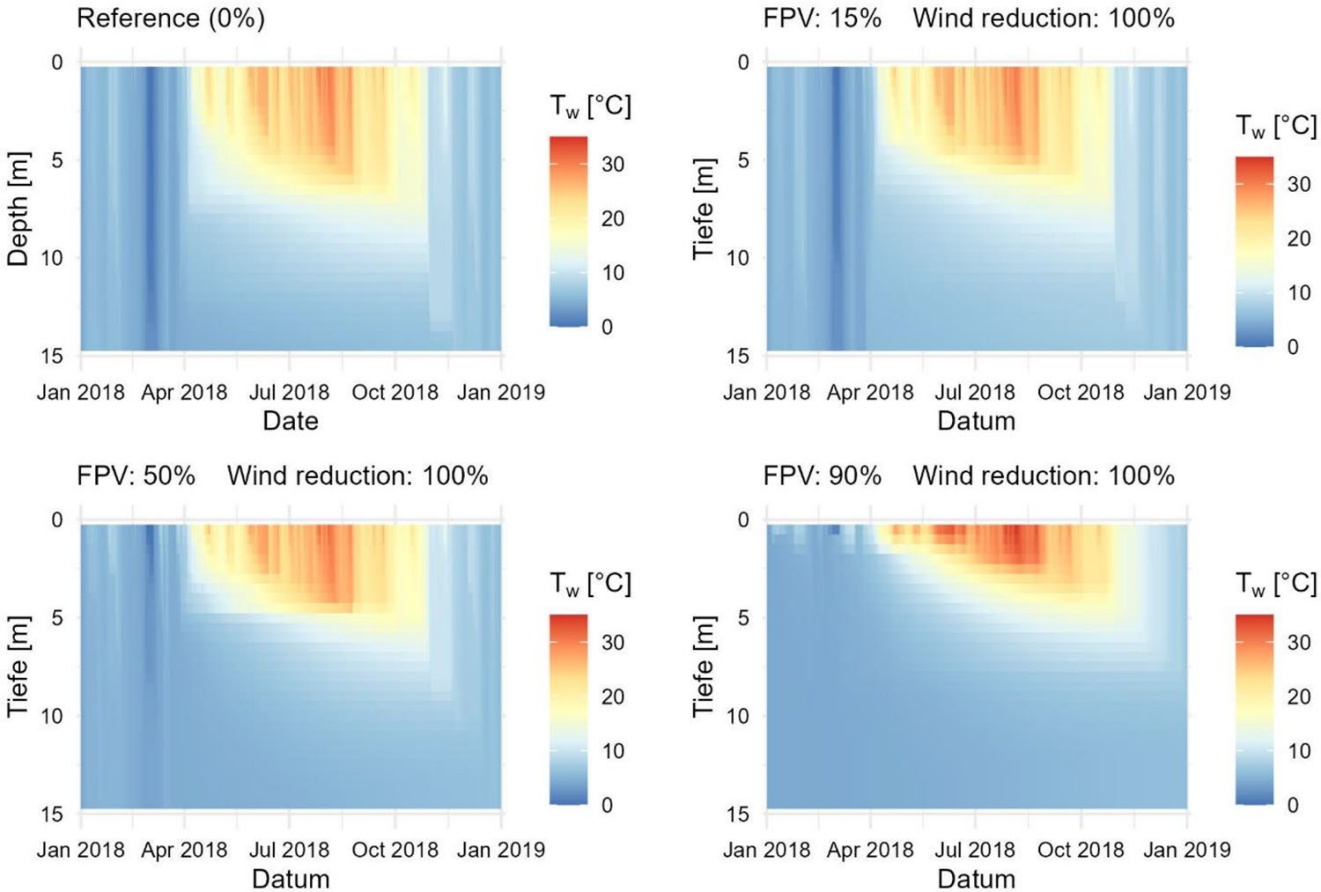
Influence of a change in wind reduction by FPV on water temperatures and thermal stratification in the lake for the year 2018. The wind reduction is increased from the measured 23% to 100%.

Figure 12 shows the impact of a maximum wind reduction on water temperatures in 2018. Here, the wind reduction is increased from 23 to 100%. As in the previous simulations, the irradiance reduction remains at 73%. It can be observed that the surface water temperatures of the 90% scenario are significantly higher compared to Fig. 10. The dissolution of the summer stratification also shifts further back in the year. For lower FPV occupancies, the changes are smaller. However, increased water temperatures in the epilimnion can also be seen here.

**Figure 13:**
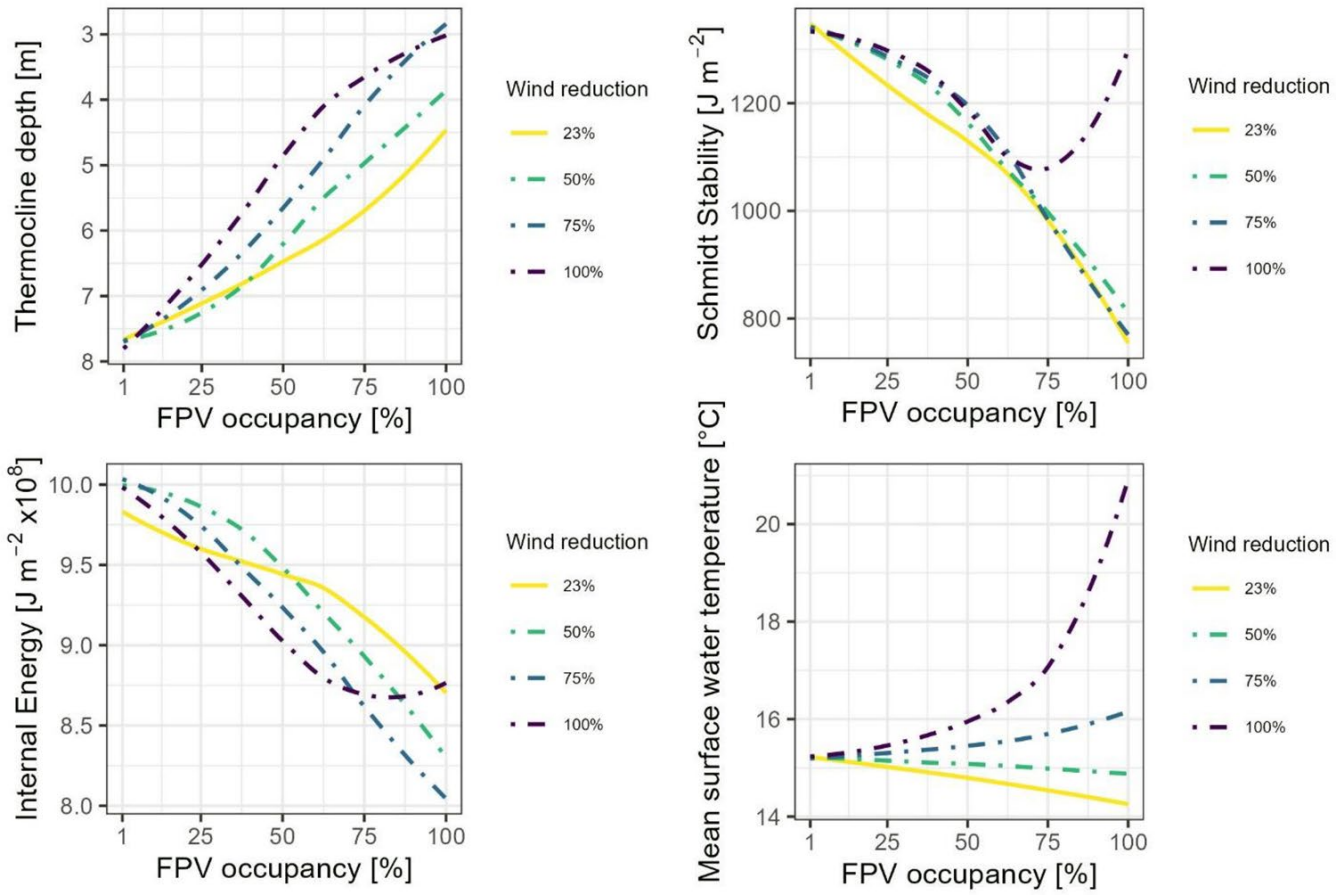
Sensitivity analysis of wind reduction through FPV. Four scenarios with different wind reductions (23%, 50%, 75% and 100%) are shown. The solid line indicates the scenario with measured reductions for irradiance (73%) and wind (23%). The dot-dashed lines indicate an adjustment of the wind reduction, while the irradiation reduction remains constant. The effect on variables characterising the thermal properties of the lake, such as thermocline depth, Schmidt stability, internal energy and surface water temperature, can be observed*.*

In a next step, the effect of different wind reduction scenarios on the variables thermocline depth, S_T_, E and mean surface water temperature is simulated (Fig. 13). FPV occupancy is increased stepwise from 1 to 100%. The wind reductions are 23%, 50%, 75% and 100%. The irradiation reduction remains constant at 73%. The solid line reflects the scenario based on the measured values, while the dot-dashed lines refer to scenarios with increased wind reduction. For all variables considered, non-linear curves can be observed with increasing FPV occupancy. Regarding the thermocline depth, all scenarios show a shift of the thermocline to lower depths with increasing FPV occupancy. While the thermocline of the 100% scenario initially experiences a strong upward offset, this effect flattens out with increasing FPV occupancy. The opposite effect can be observed in the 23% scenario.

S_T_ initially decreases faster in the 23% scenario than in the other scenarios. From a FPV occupancy of approx. 60%, the curves converge. While S_T_ continues to decrease for the wind reductions of 23%, 50% and 75%, it increases again for the 100% scenario to values comparable to the initial stability.

E is initially lowest in the 23% scenario at low FPV occupancy, but decreases less with increasing FPV occupancy compared to the other scenarios. From approx. 60% FPV occupancy, the reduction becomes stronger in the 23% scenario, while at a wind reduction of 100%, E increases again and shows the highest value at maximum FPV occupancy. The 75% scenario shows the lowest value.

The mean surface water temperature decreases in the scenarios with a wind reduction of 23% and 50% with increasing FPV occupancy. However, an increase in surface water temperatures can be observed in the 75% and 100% scenarios. this is particularly pronounced in the 100% scenario and shows an exponential trend.

Based on the sensitivity analysis of the wind reduction, it can be observed that the impact of FPV on the near-surface wind speed can have a considerable effect on the thermal properties of the lake. While the measured reductions in irradiance (73%) and wind (23%) imply a counteracting effect to climate change, high wind reductions can have a stabilising effect on the stratification and significantly increase the surface water temperatures in extreme years such as 2018. This, in turn, could further amplify the expected effects of climate change.

Therefore, there is a need for further research into monitoring the PV-water interaction of FPV systems. These findings can contribute to designing the systems in order to achieve beneficial effects for the water bodies with regard to climate change.

## Conclusions

Overall, the present study suggests that the current FPV system with 2% occupancy hardly causes any changes to the thermal characteristics of Lake Maiwald. This becomes evident in small variations in average T_w_ and S_T_. However, a closer look at temporal dynamics of T_w_ and related energy balance suggests that FPV diminishes the variability in surface water temperatures and increases the heat storage capacity of the lake. In general, FPV retards the warming of the water layers, an effect that increases with rising GHI and T_air_. In addition, FPV delays lake water cooling by reducing its heat emissions, particularly during nights after high daytime GHI. The daily minimum and the maximum of the heat storage are delayed on average by two hours for FPV. In the study period from mid-July to mid-October, the highest average cooling during the day with −0.443 °C is observed in July, while the least occurred in October with −0.106 °C. At night, this is significantly less pronounced and turned into less heat emission below FPV in October (+ 0.016 °C). The impact of FPV is highest in the near-surface water layer (0 to 5 m) and can hardly be detected in deeper layers from 5 to 10 m (p = 0.007). Also, a reduction in near-surface wind speed is evident, but relatively small, so that the FPV system on Lake Maiwald can be classified as a wind-dominant, because near-surface lateral windflow is less reduced (by 23%) than irradiation into the lake (by 73%).

Model simulations of the extreme year 2018, incorporating these measured reductions for irradiation and wind, indicate that FPV counteracts a more stable and longer stratification that might be induced by climate change. However, a sensitivity analysis of the wind reduction by FPV showed considerable effects on the thermal properties of the lake. Therefore, high wind reductions can have a opposite effect and could further amplify the expected effects of climate change.

The present study suggests that Lake Maiwald represents a preferential FPV site, with distinct resilience with respect to FPV impacts. However, there is still further research needed on the PV-water interaction, the effects on biochemical processes in the water body, as well as on tuning FPV plants to maximum yields with minimum effects on lake ecosystems. Overall, hydro-ecological impact assessments should be an integral part for sustainable integration of FPV systems. Such studies also widen the understanding of FPV impacts on water bodies and help to maximise synergy effects of renewable electricity generation and ecosystem preservation in the face of climate change.

## Supplementary Information

Supplementary Information.

## Data Availability

All data generated or analysed during this study are included in this published article [and its supplementary information files].
