# Correction: The impact of floating photovoltaic power plants on lake water temperature and stratification

**DOI:** 10.1038/s41598-026-37857-5

**Published:** 2026-02-05

**Authors:** Konstantin Ilgen, Dirk Schindler, Stefan Wieland, Jens Lange

**Affiliations:** 1 Fraunhofer Institute for Solar Energy Systems ISE, Heidenhofstr. 2, 79110 Freiburg, Baden‑Württemberg Germany; 2 Hydrology, Faculty of Environment and Natural Resources, University of Freiburg, Freiburg, Baden‑Württemberg Germany; 3 Environmental Meteorology, Faculty of Environment and Natural Resources, University of Freiburg, Freiburg, Baden‑Württemberg Germany

Correction to: *Scientific Reports* 10.1038/s41598-023-34751-2, published online 16 May 2023

The original version of this Article contained errors in the derivation of the radiation and wind stress per unit area, in the Materials and methods section.

As a result, in the Materials and methods.

“

4a $${sw}_{aw}={sw}_{f}\left(1-\frac{{A}_{FPV}}{{A}_{S}}\left[{sw}_{f}-{sw}_{r}\right]\right)$$

4b $${w}_{aw}={w}_{f}\left(1-\frac{{A}_{FPV}}{{A}_{S}}\left[{w}_{f}-{w}_{r}\right]\right)$$”

now reads:

“

4a $${sw}_{aw}={sw}_{f}\left(1-\frac{{A}_{FPV}}{{A}_{S}}{sw}_{r}\right)$$

4b $${w}_{aw}={w}_{f}\left(1-\frac{{A}_{FPV}}{{A}_{S}}{w}_{r}\right).$$”

This error is purely notational; all results in the paper were obtained through the application of the correct (latter) Equations (4a)–(4b).

The original Article has been corrected.

